# Supplementary figures and images for: AMP activated kinase negatively regulates hepatic Fetuin-A via p38 MAPK-C/EBPβ/E3 Ubiquitin Ligase Signaling pathway
Source: PLoS One. 2022 May 6;17(5):e0266472. doi: 10.1371/journal.pone.0266472 (PMC9075660; doi:10.1371/journal.pone.0266472)

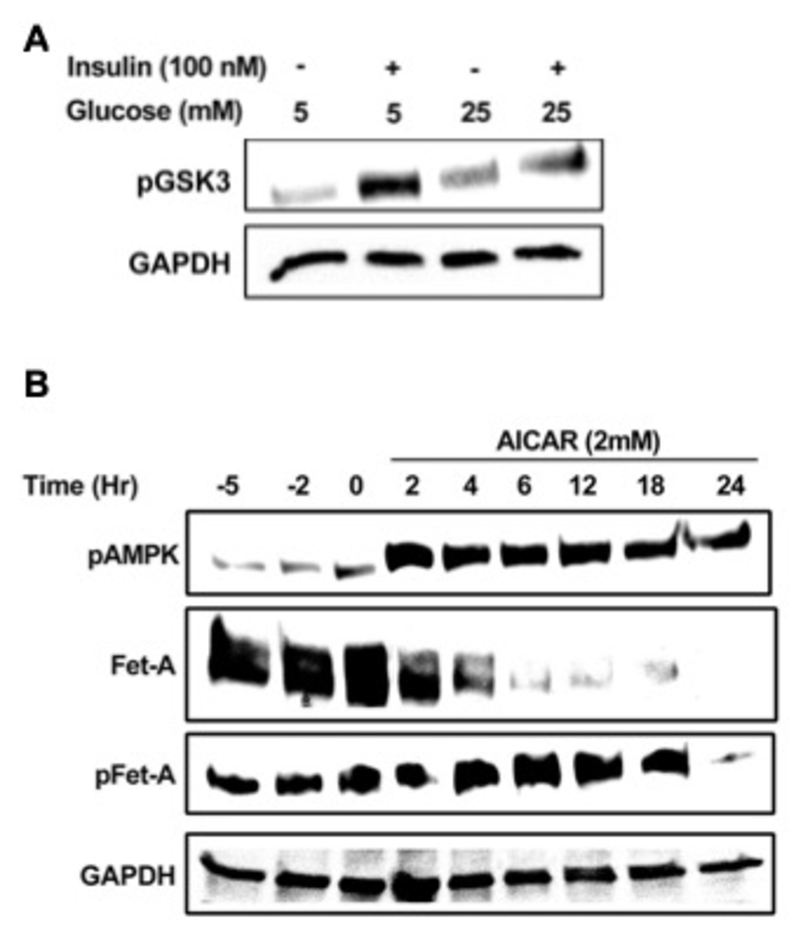

Supplement: S1 Fig — HepG2 cells were incubated in low (5 mM) or high glucose (25 mM) media for 12 h followed by insulin treatment for 15 min and cell lysate were subjected to immunoblotting for GSK3 phosphorylation status. [B] The effect of AICAR (2 mM) on HepG2 cells under high glucose condition at different periods was observed by Western blot analysis. (TIF) [file pone.0266472.s001.tif]
