# Supplementary material for: AMP activated kinase negatively regulates hepatic Fetuin-A via p38 MAPK-C/EBPβ/E3 Ubiquitin Ligase Signaling pathway
Source: PLoS One. 2022 May 6;17(5):e0266472. doi: 10.1371/journal.pone.0266472 (PMC9075660; doi:10.1371/journal.pone.0266472)

Figure 1A

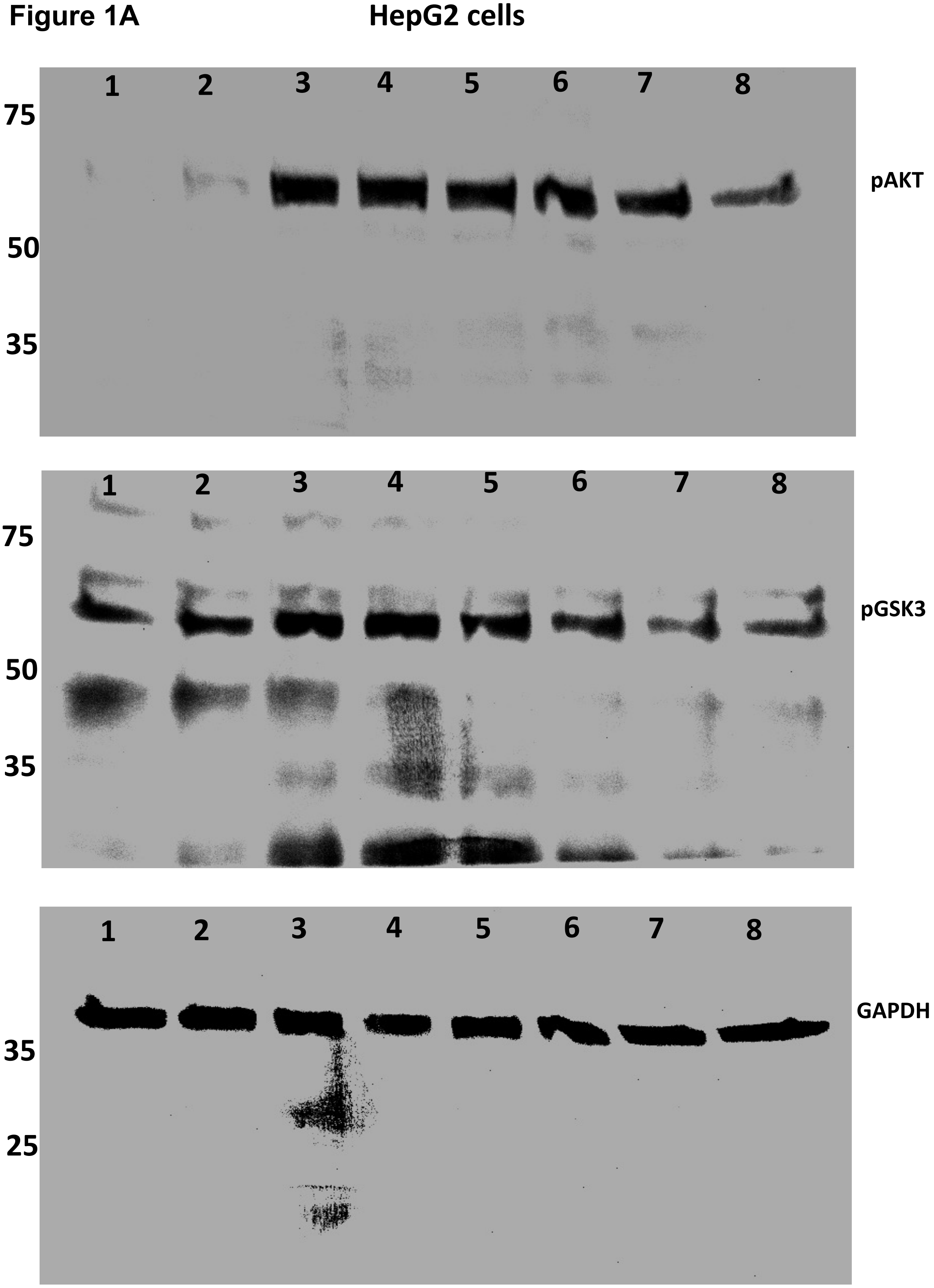
HepG2 cells
1 2 3 4 5 6 7 8
75
50
35
pAKT
1 2 3 4 5 6 7 8
75
50
35
pGSK3
1 2 3 4 5 6 7 8
35
25
GAPDH

Figure 2A

HepG2 cells lysate

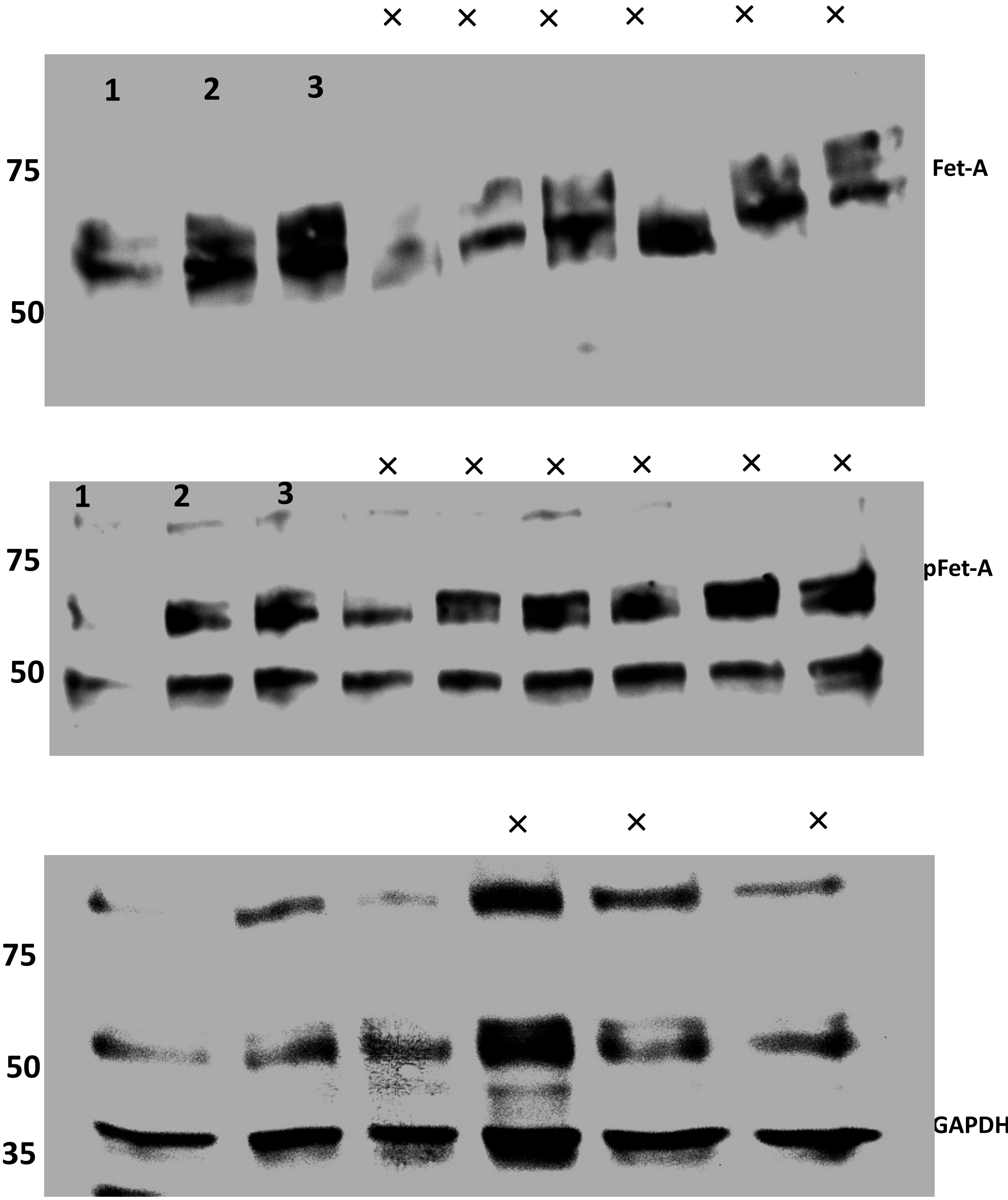

Figure 2A

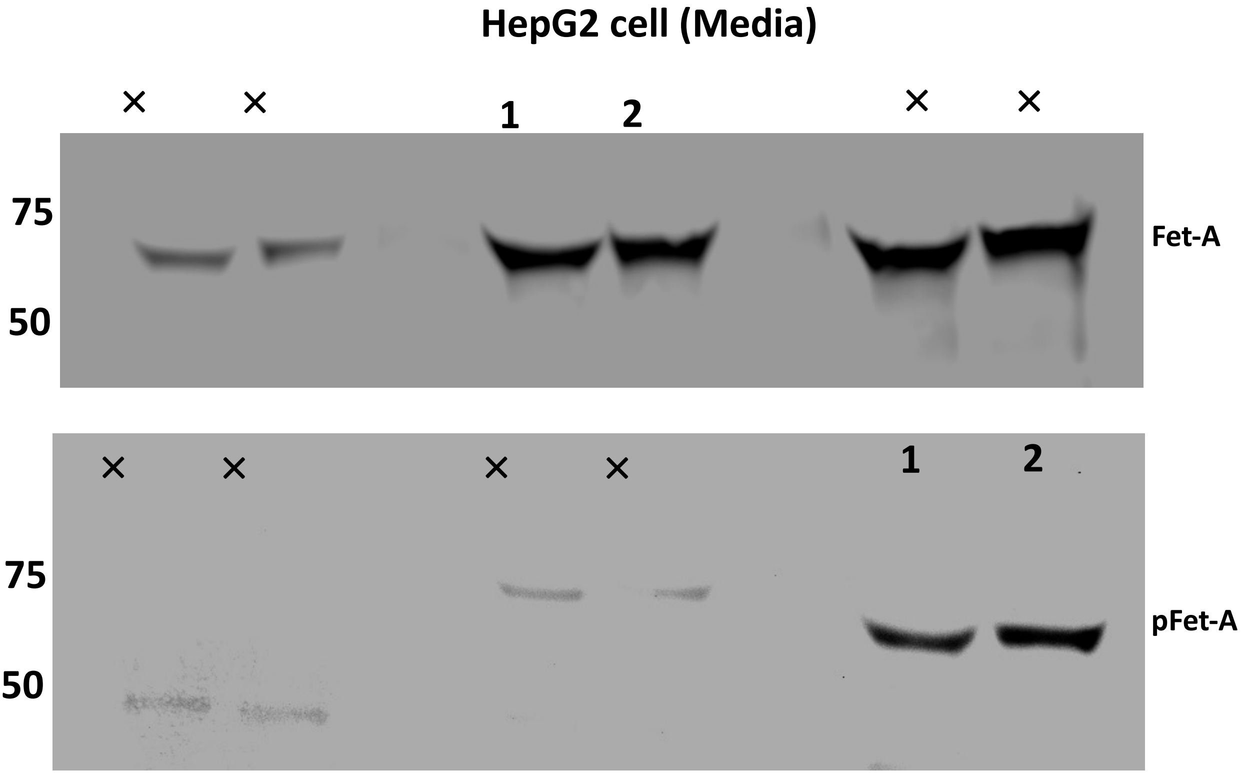

Figure 2B

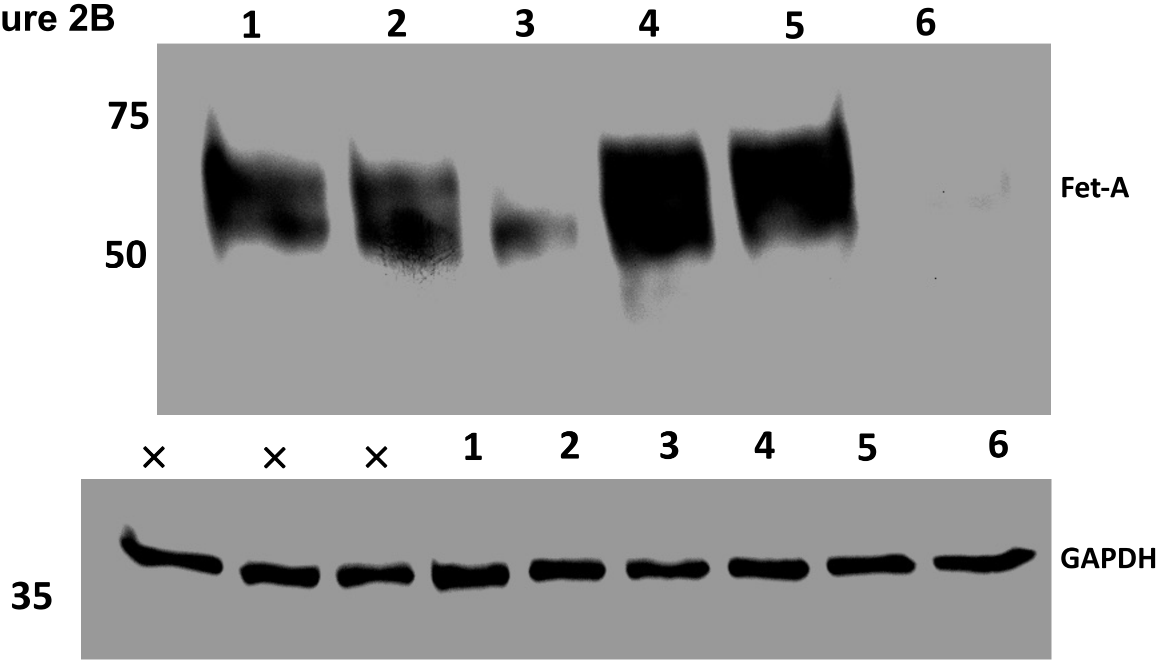

Figure 2C

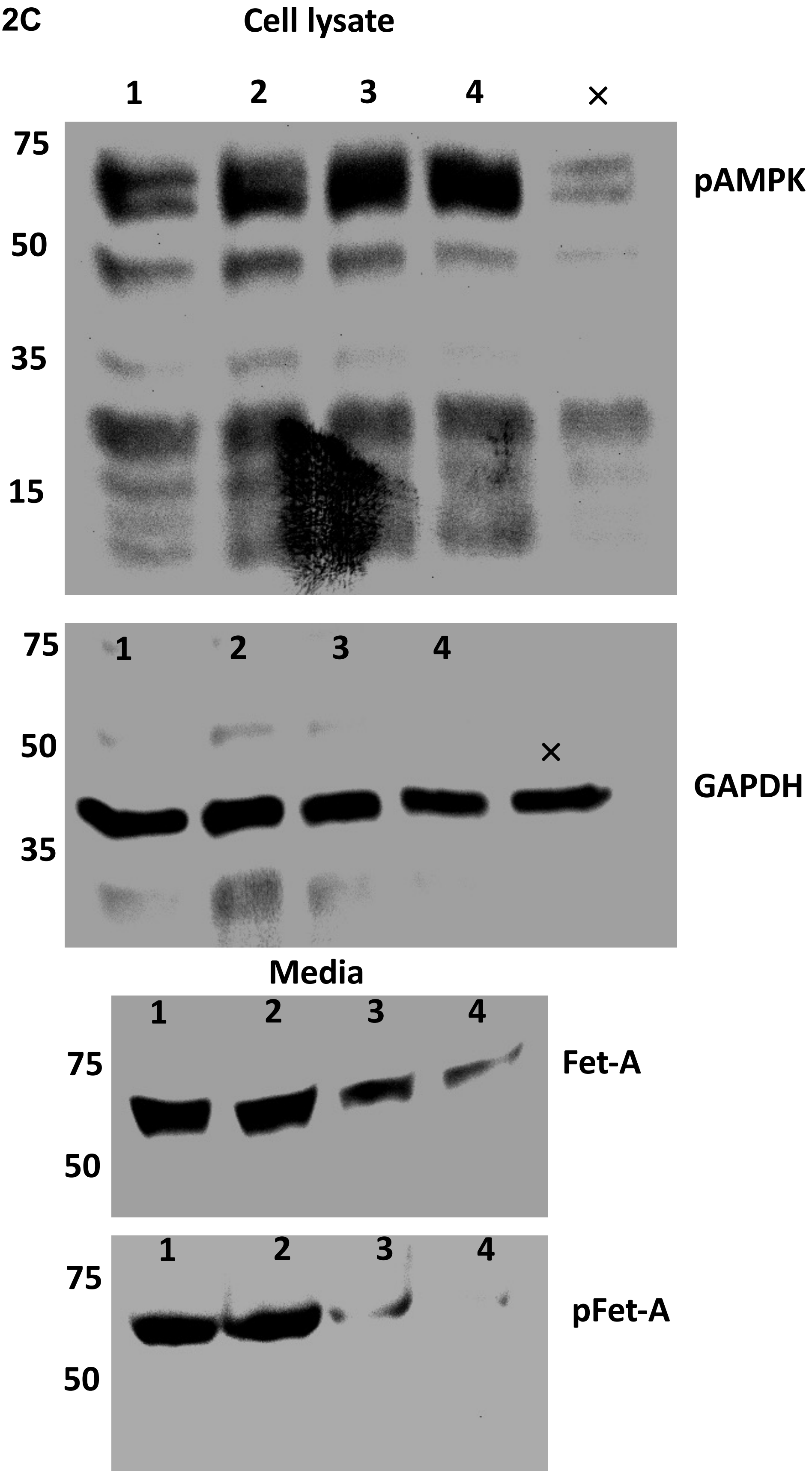
Cell lysate
1
2
3
4
×
75
50
35
15
pAMPK
75
50
35
GAPDH
Media
75
50
Fet-A
75
50
pFet-A

Figure 2G

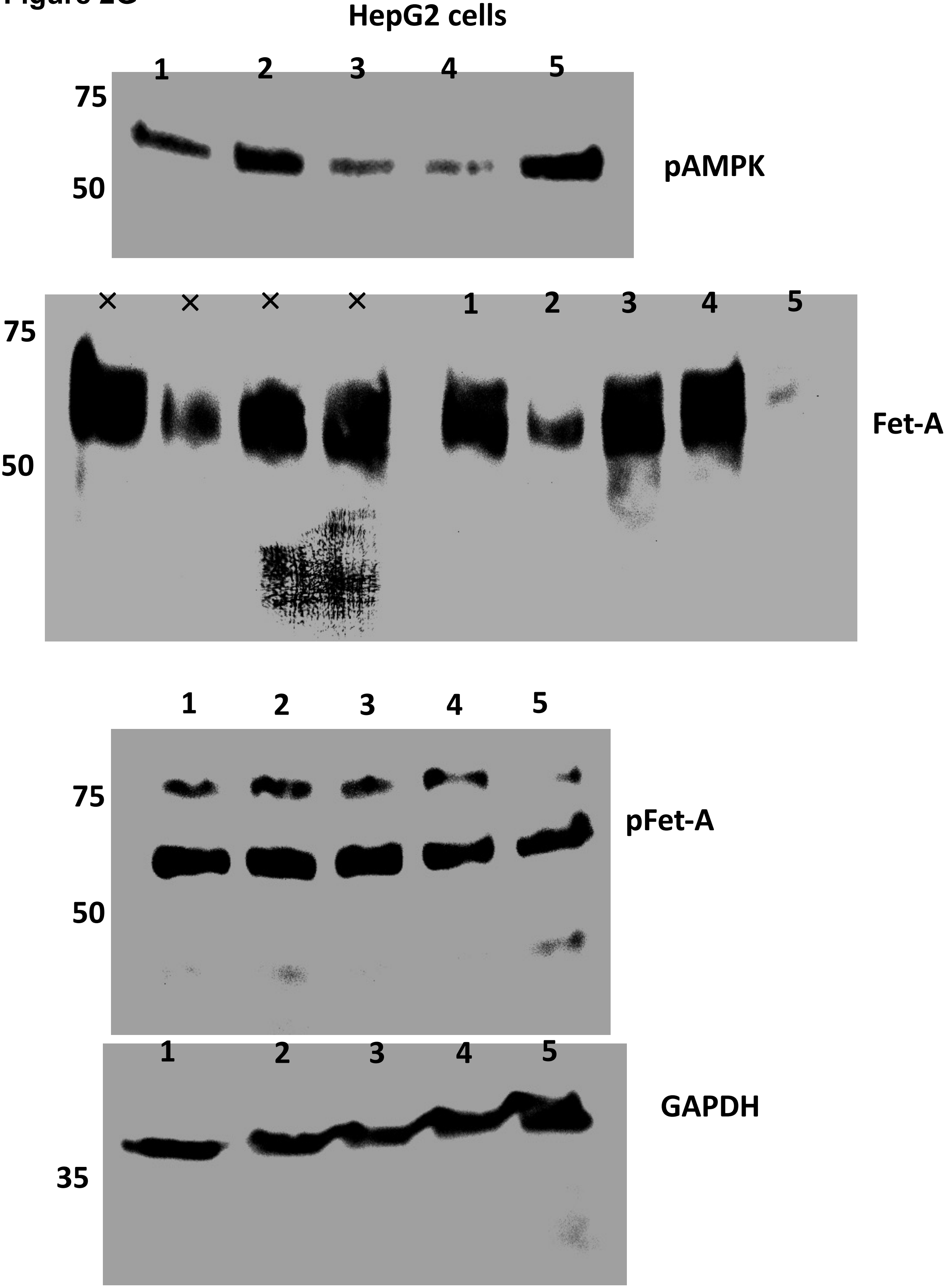
HepG2 cells
1 2 3 4 5
75
50
pAMPK
× × × × 1 2 3 4 5
75
50
Fet-A
1 2 3 4 5
75
50
pFet-A
1 2 3 4 5
35
GAPDH

Figure 3A

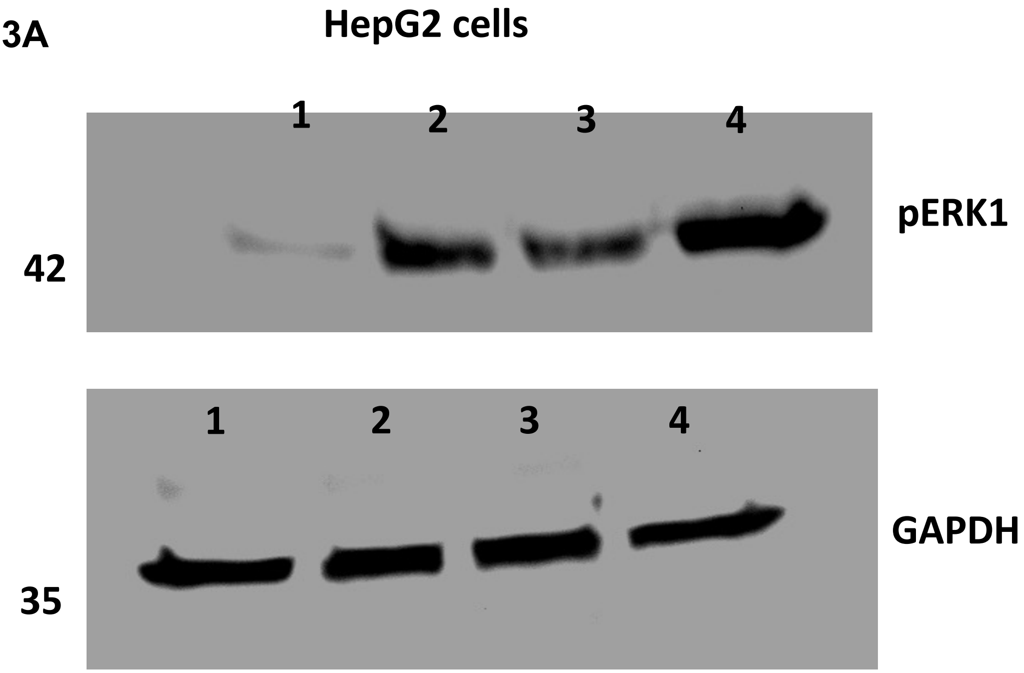

Figure 3B

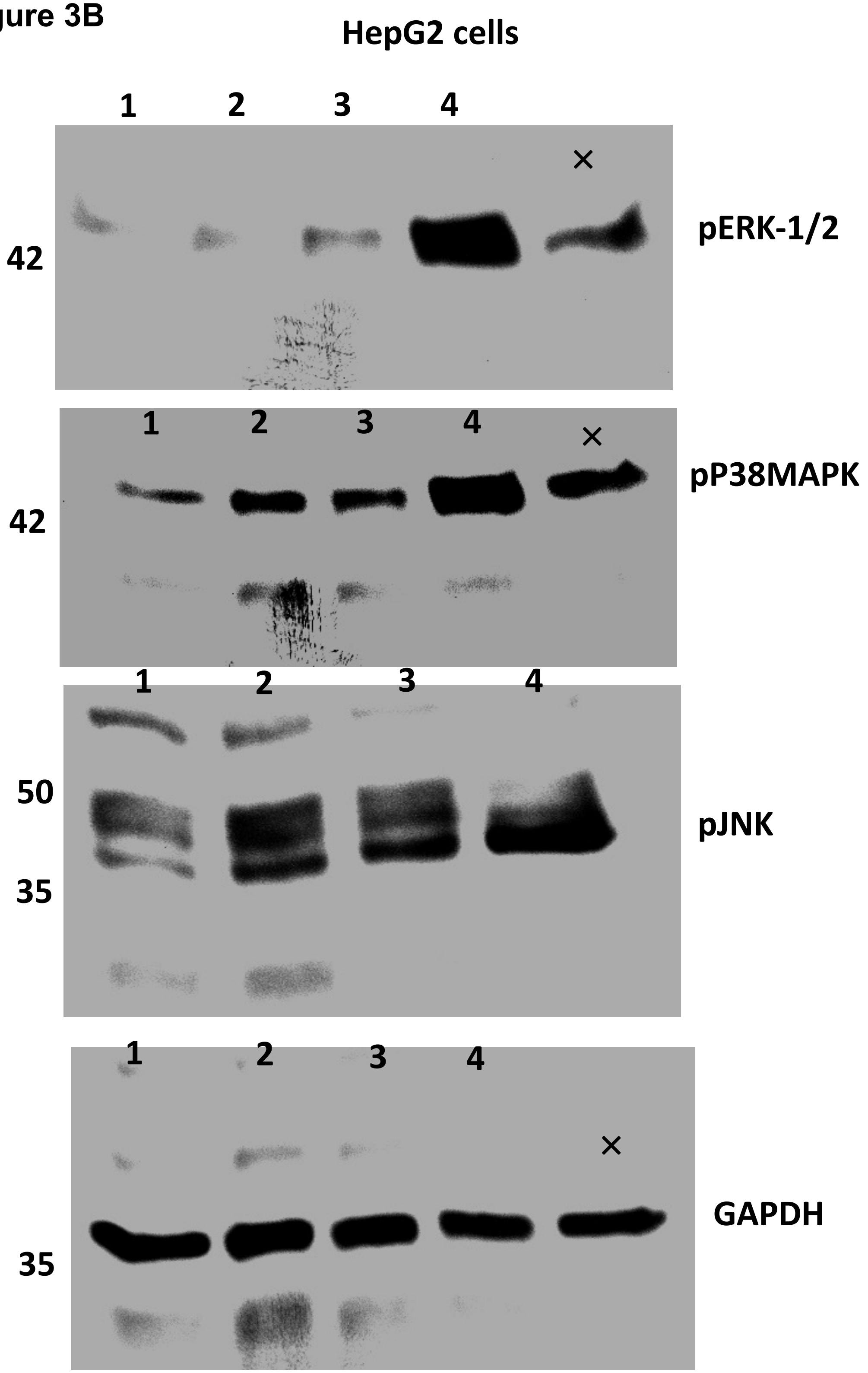

HepG2 cells
1
2
3
4
×
42
pERK-1/2
1
2
3
4
×
42
pP38MAPK
1
2
3
4
50
35
pJNK
1
2
3
4
×
35
GAPDH

Figure 3C

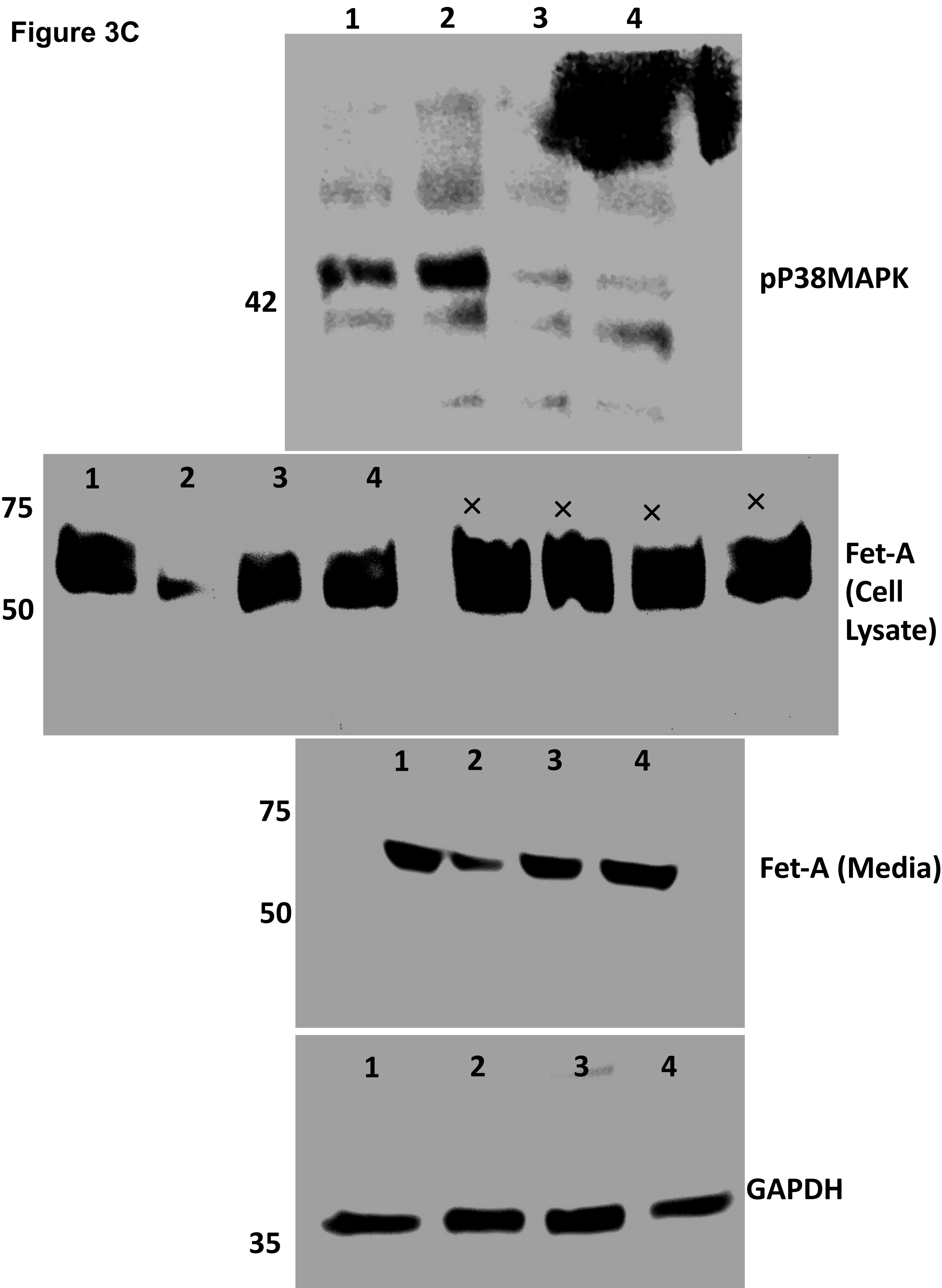
1 2 3 4
42
pP38MAPK
1 2 3 4
75
50
Fet-A (Cell Lysate)
1 2 3 4
75
50
Fet-A (Media)
1 2 3 4
GAPDH
35

Figure 3E

HepG2 cells

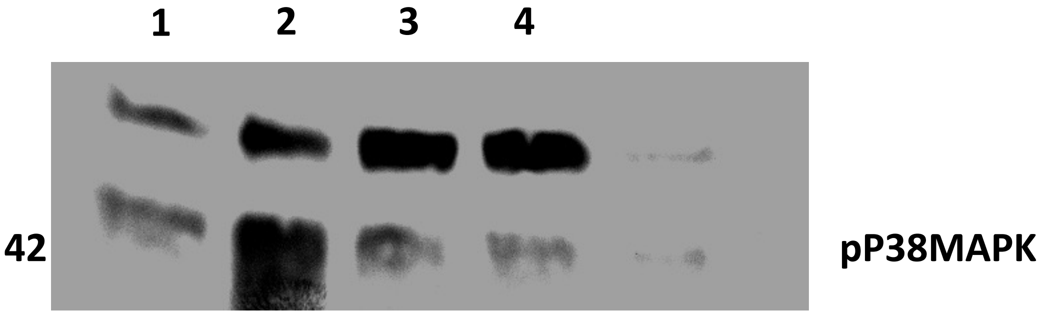

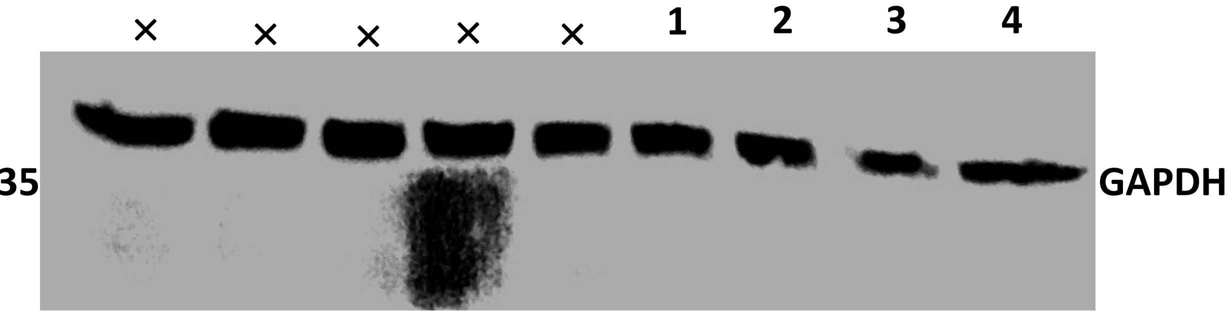

Figure 3G

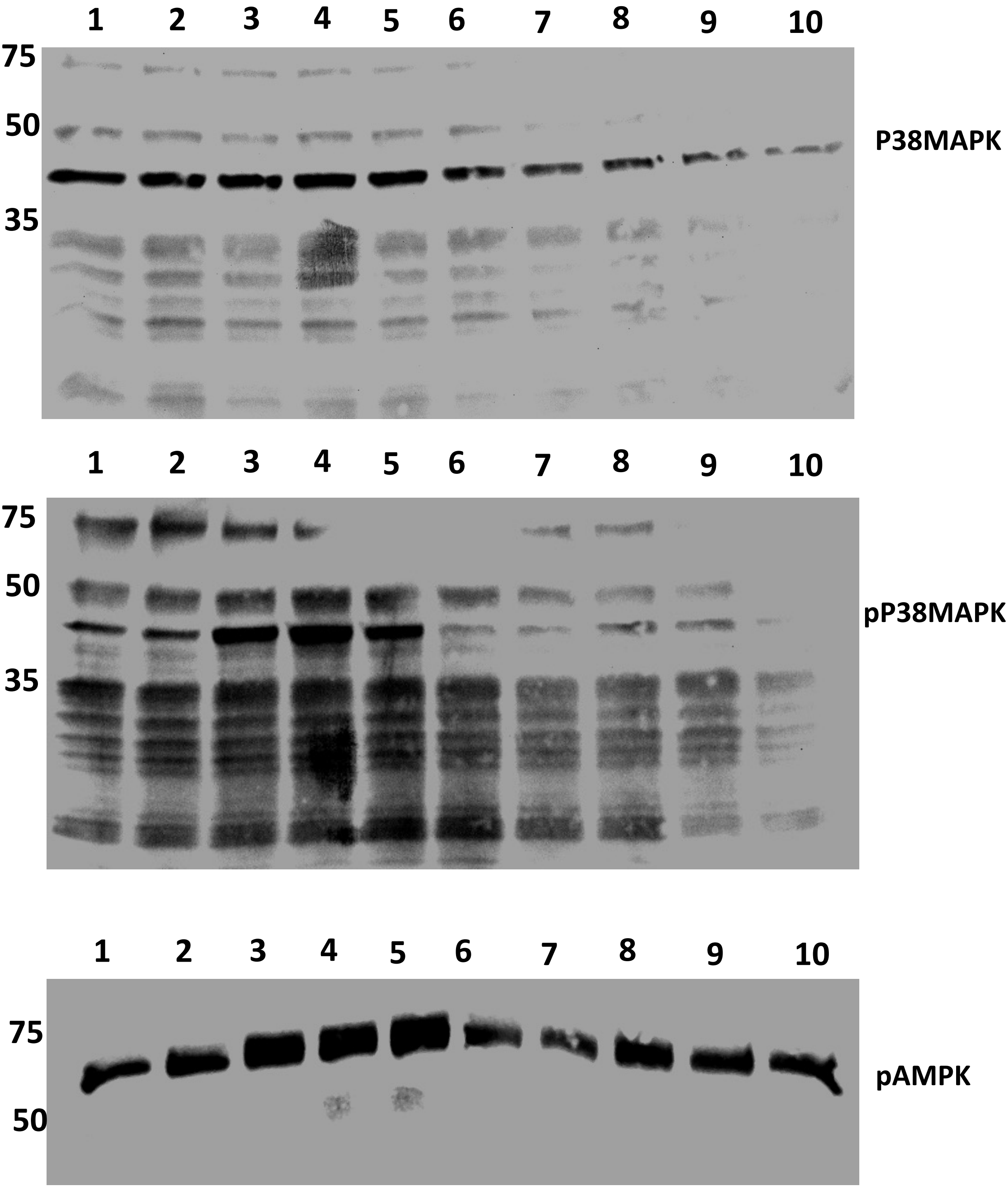
1 2 3 4 5 6 7 8 9 10
75
50
35
P38MAPK
1 2 3 4 5 6 7 8 9 10
75
50
35
pP38MAPK
1 2 3 4 5 6 7 8 9 10
75
50
pAMPK

Figure 3G

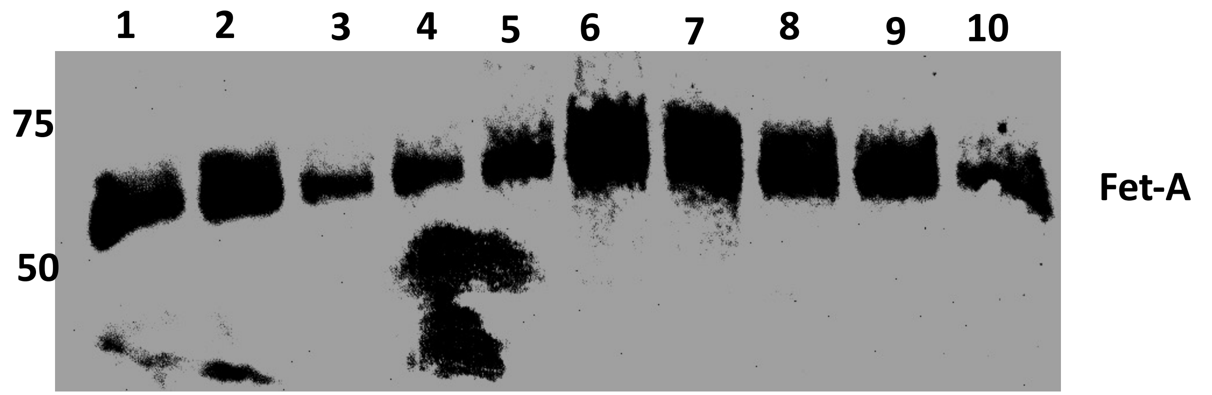

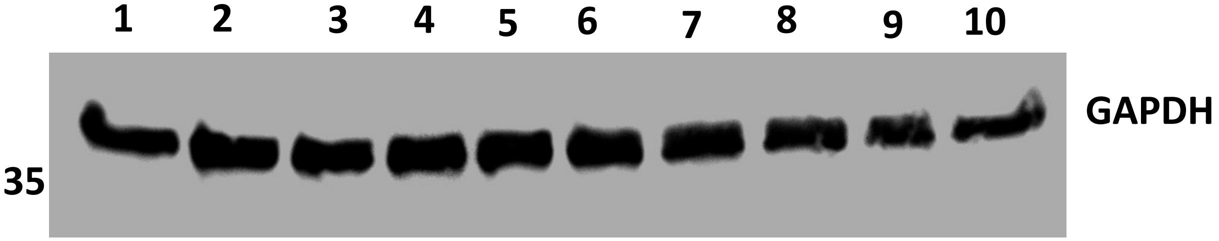

Figure 3J

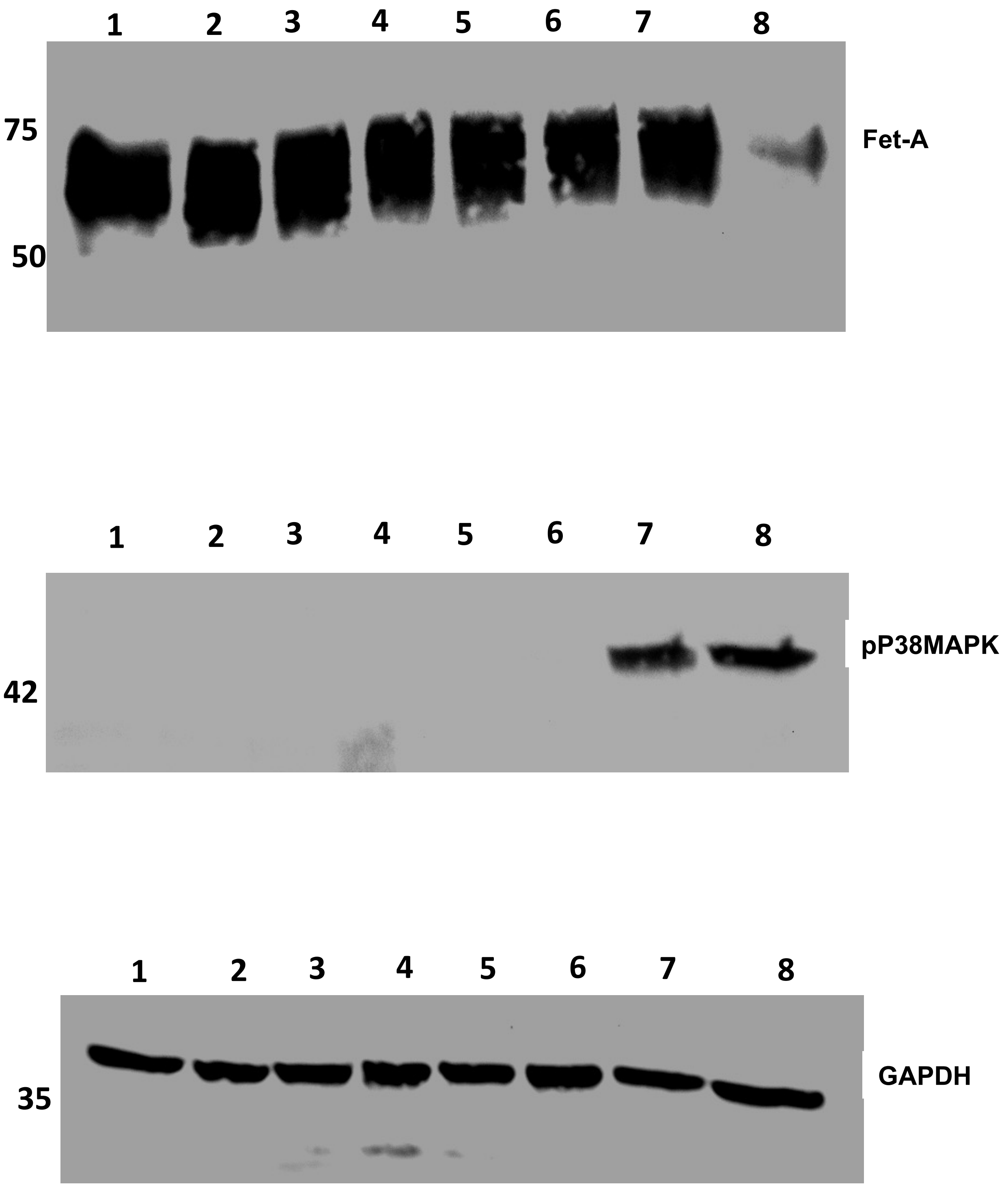

Figure 4A

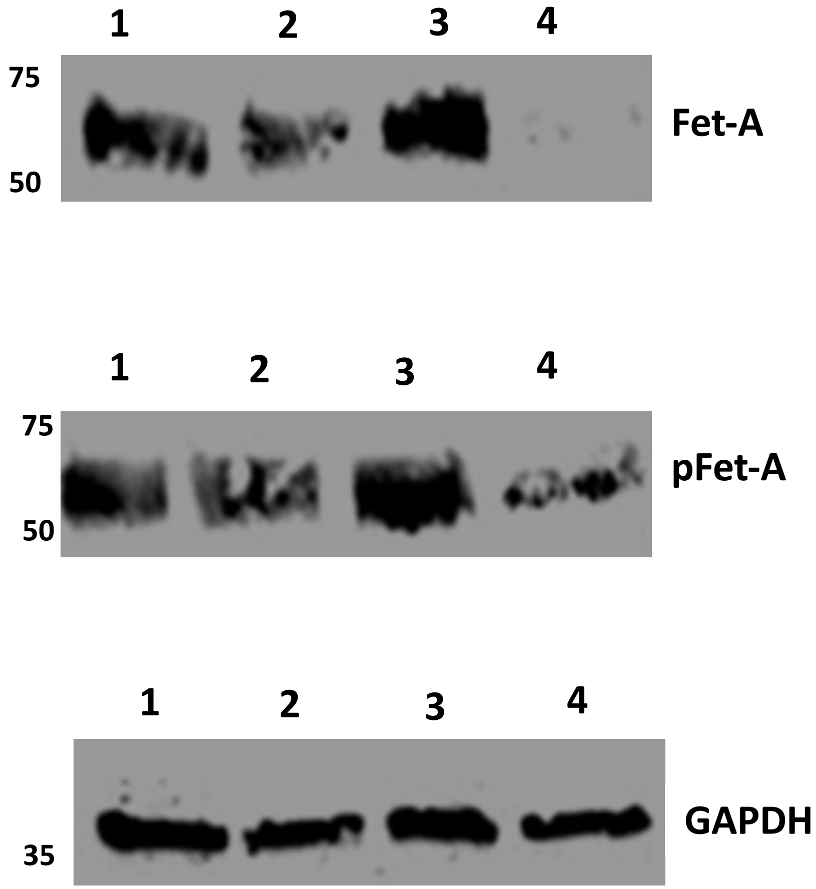

Figure 4C

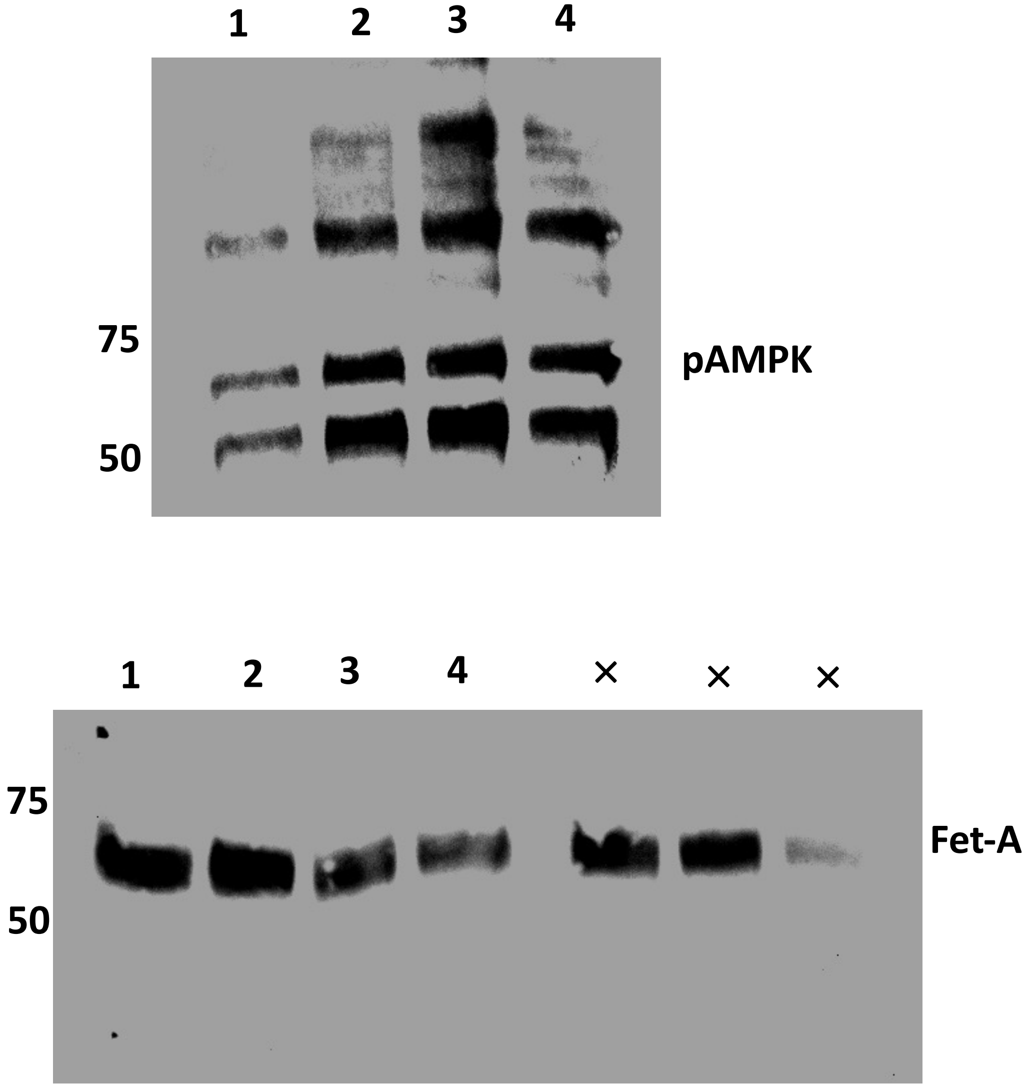

Figure 4C

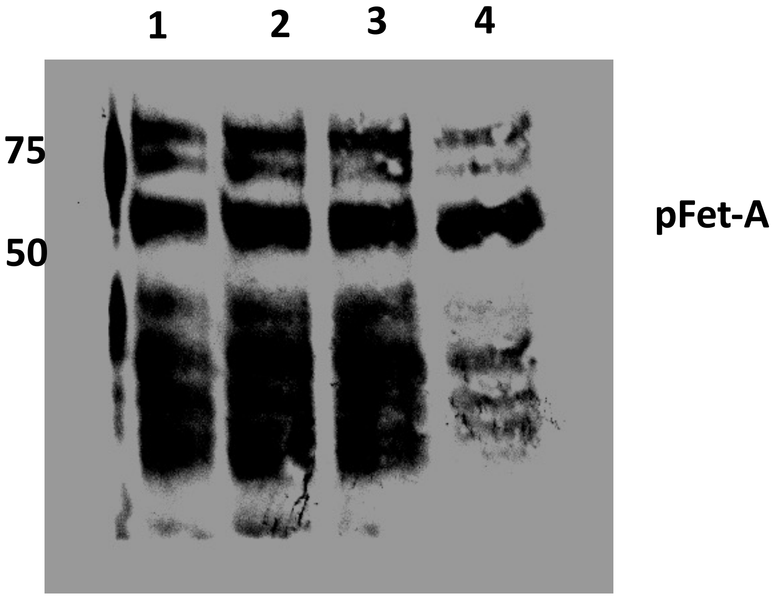

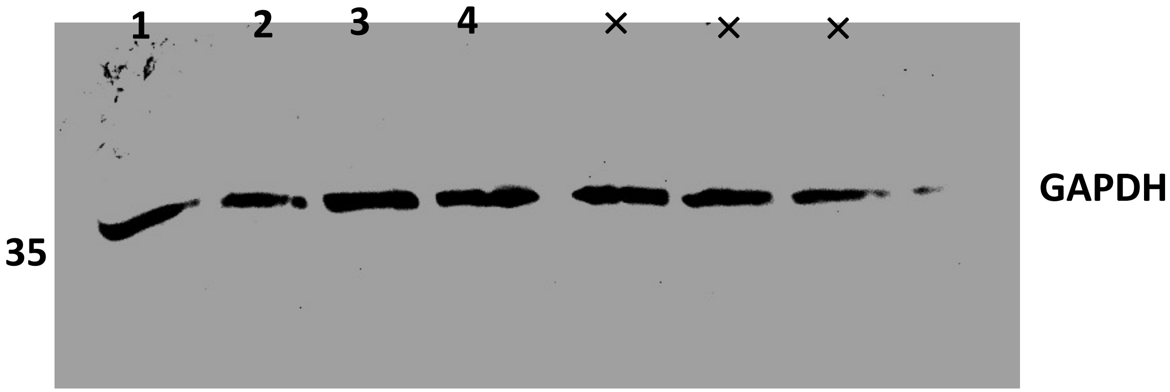

Figure 4E

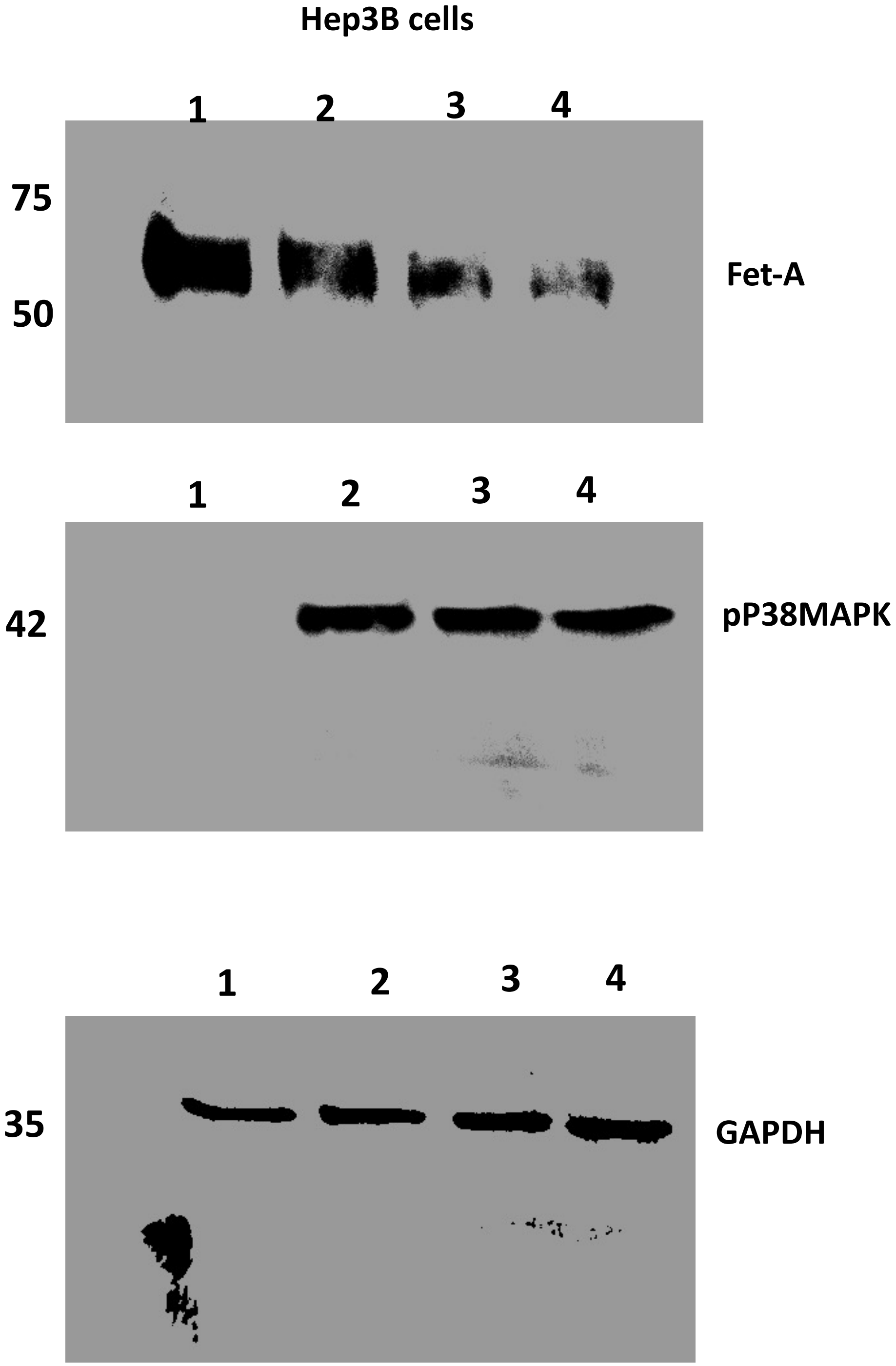

Figure 4G

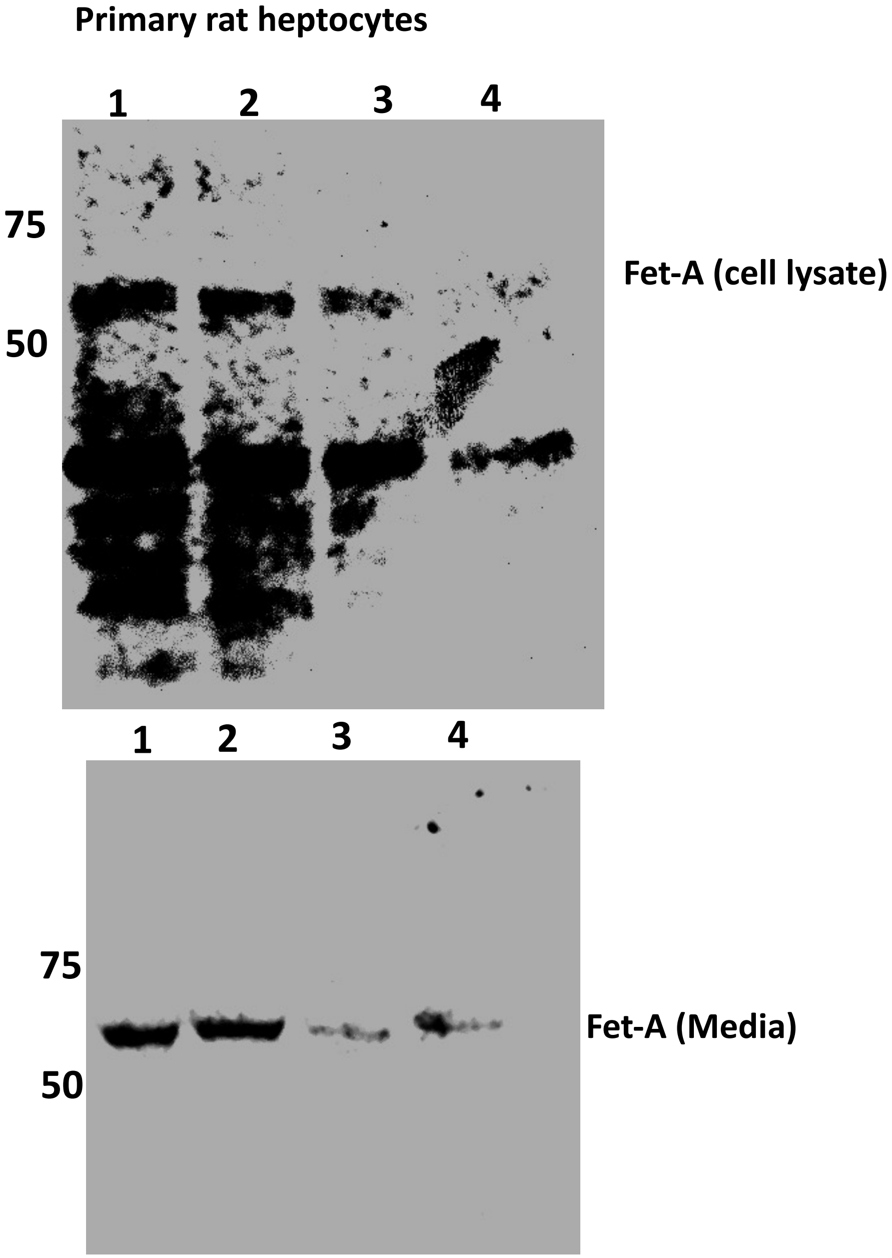
Primary rat heptocytes
1
2
3
4
75
50
Fet-A (cell lysate)
1
2
3
4
75
50
Fet-A (Media)

Figure 4G

Primary rat heptocytes

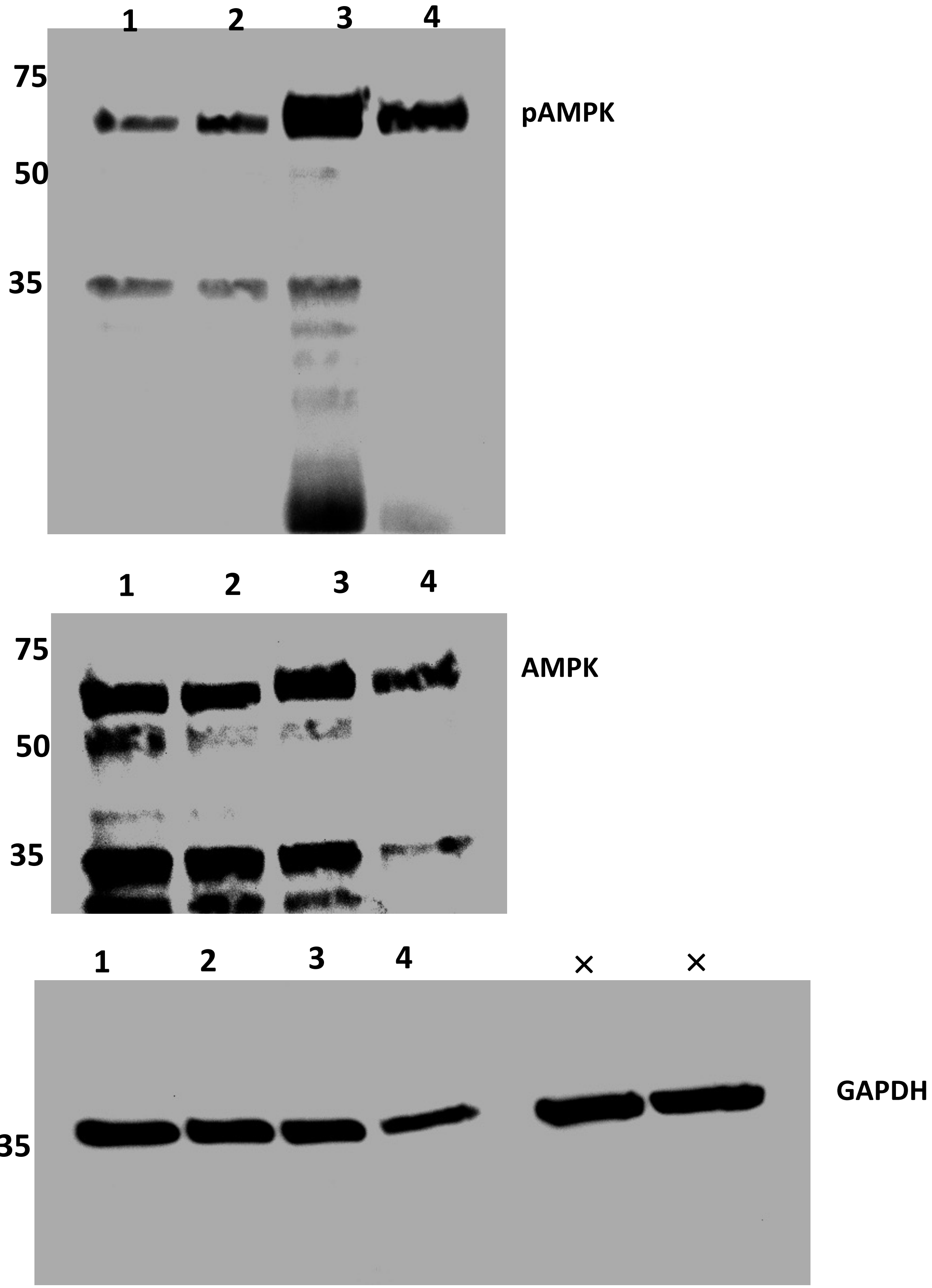

Figure 4G

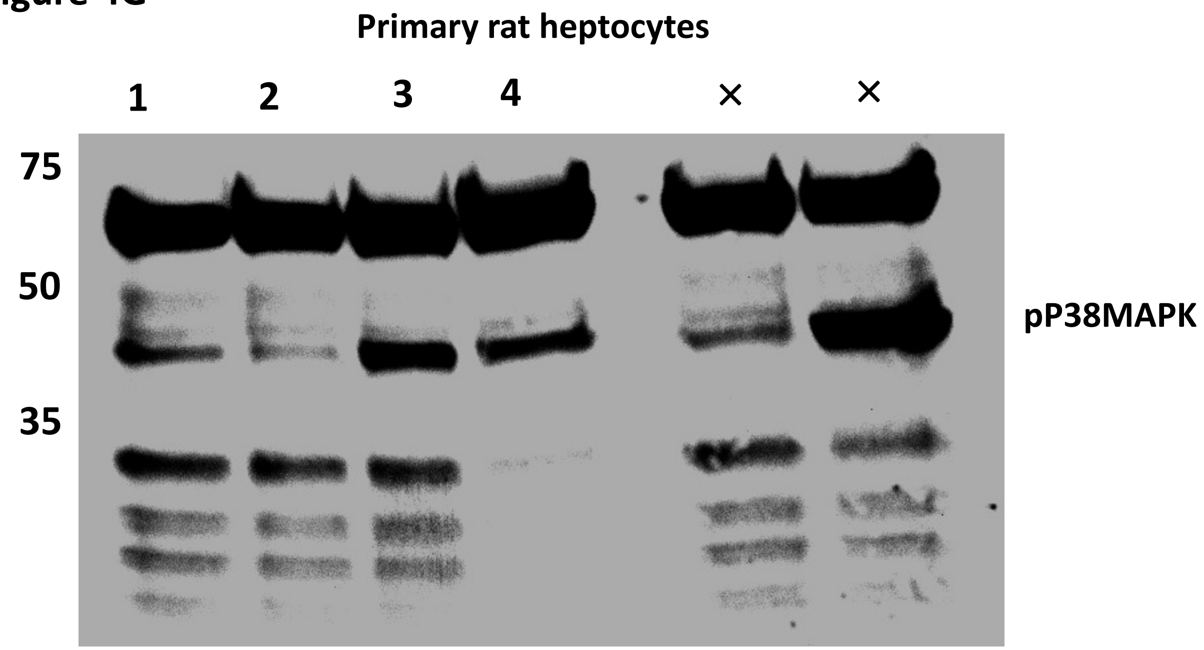

Figure 4I

Primary rat heptocytes

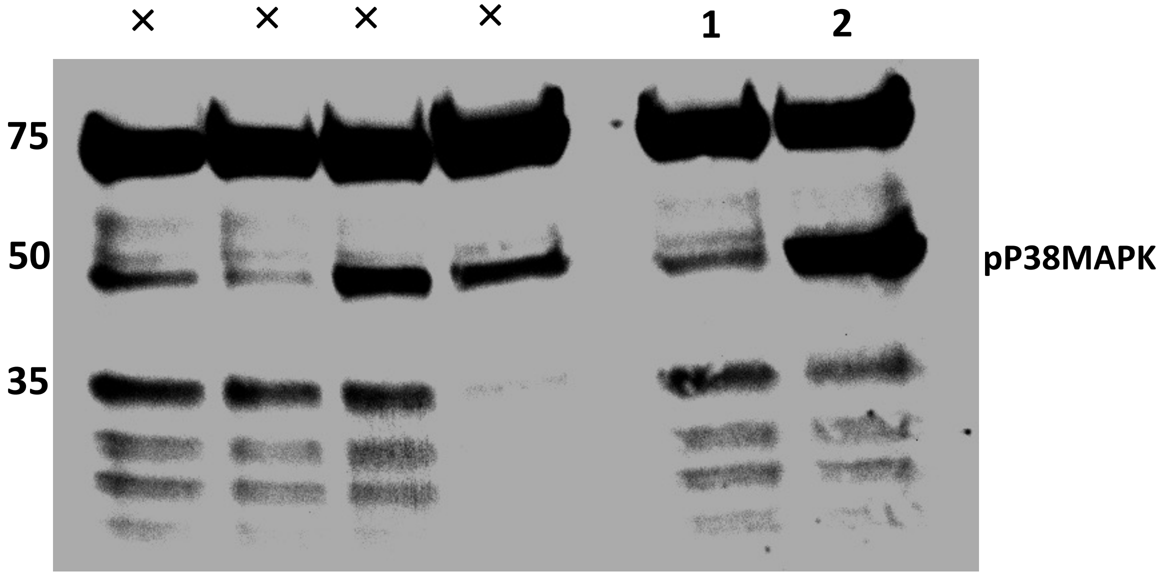

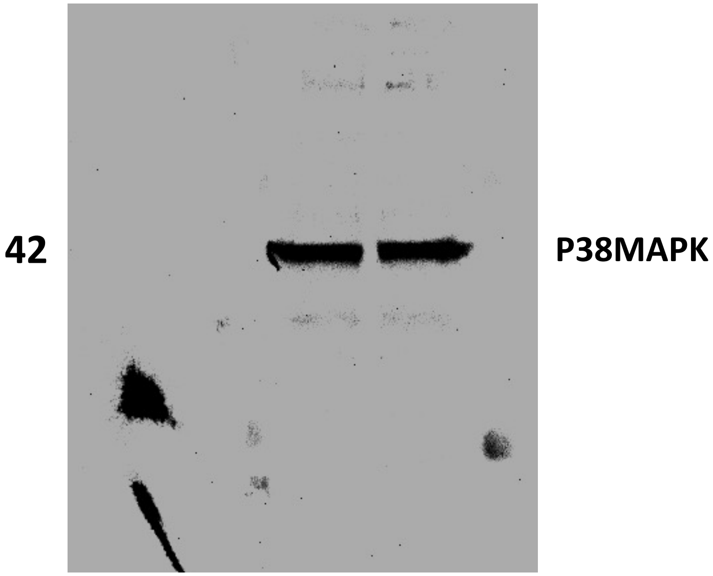

Figure 4I

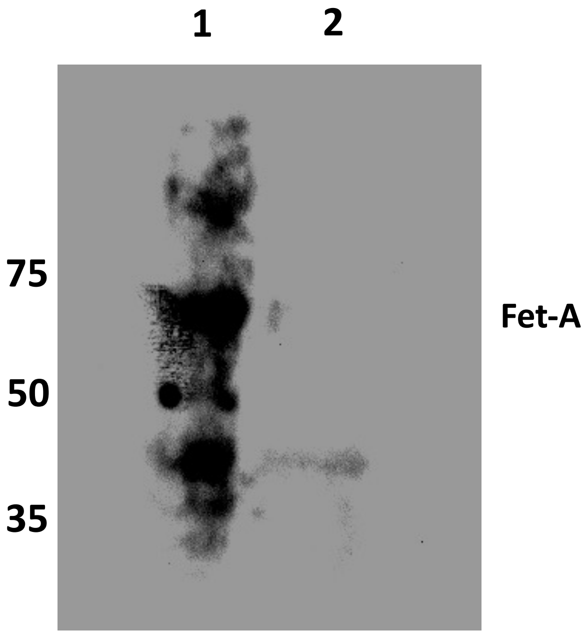

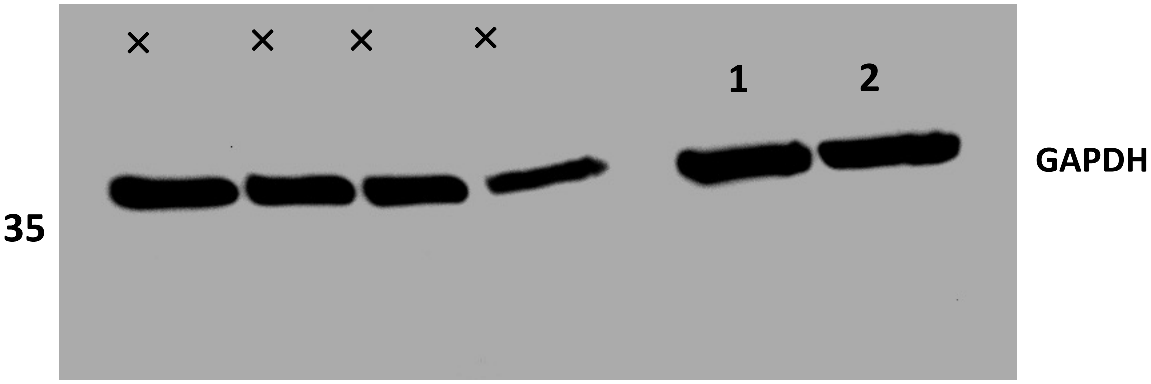

Figure 5A

HepG2 cells

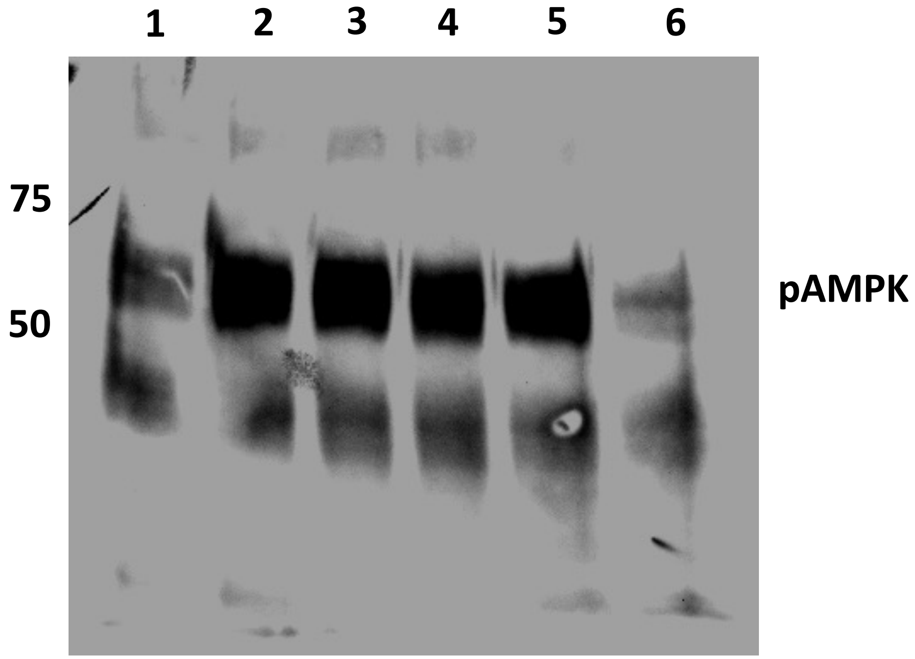

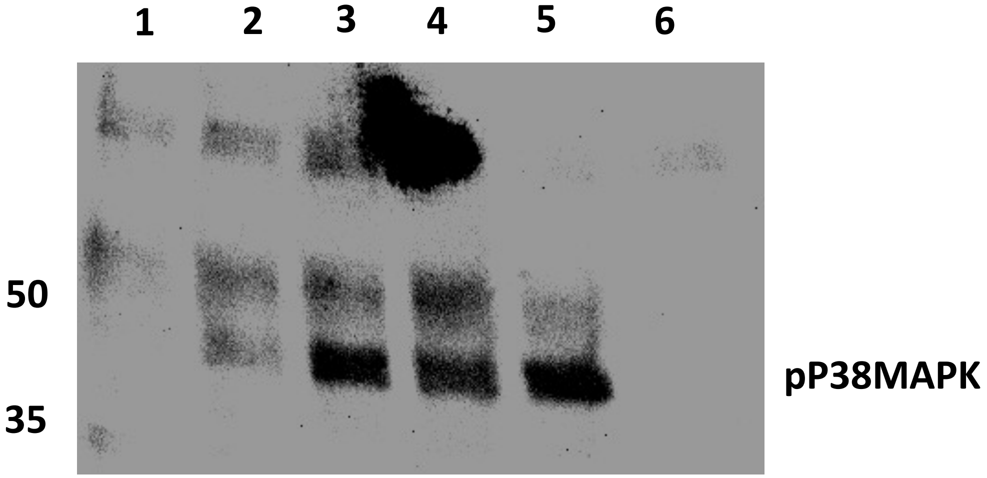

Figure 5A

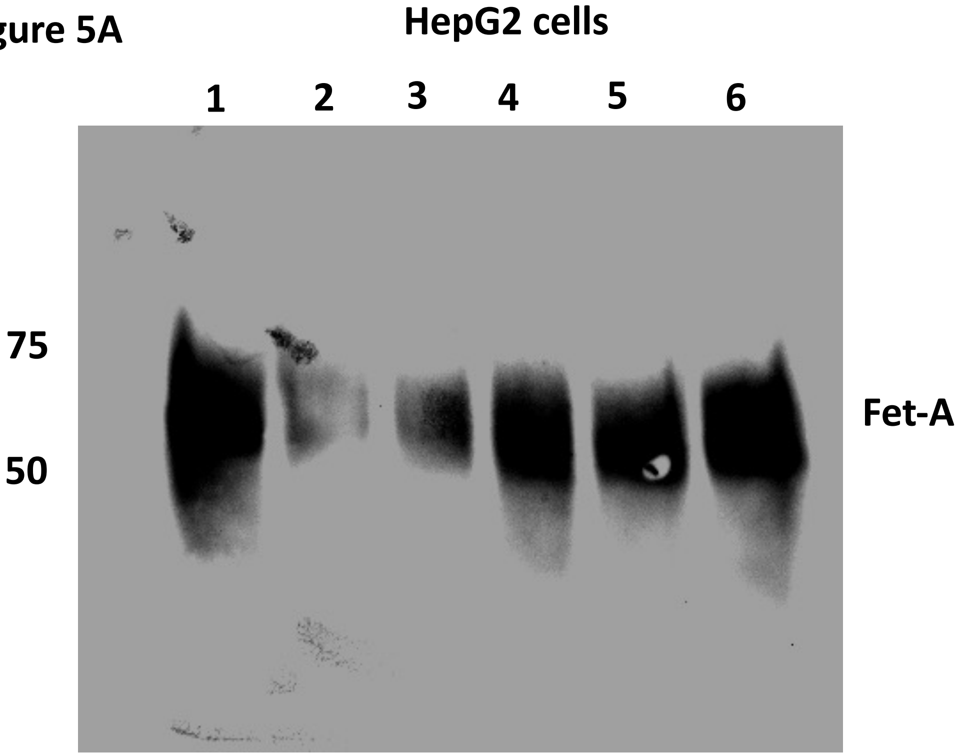

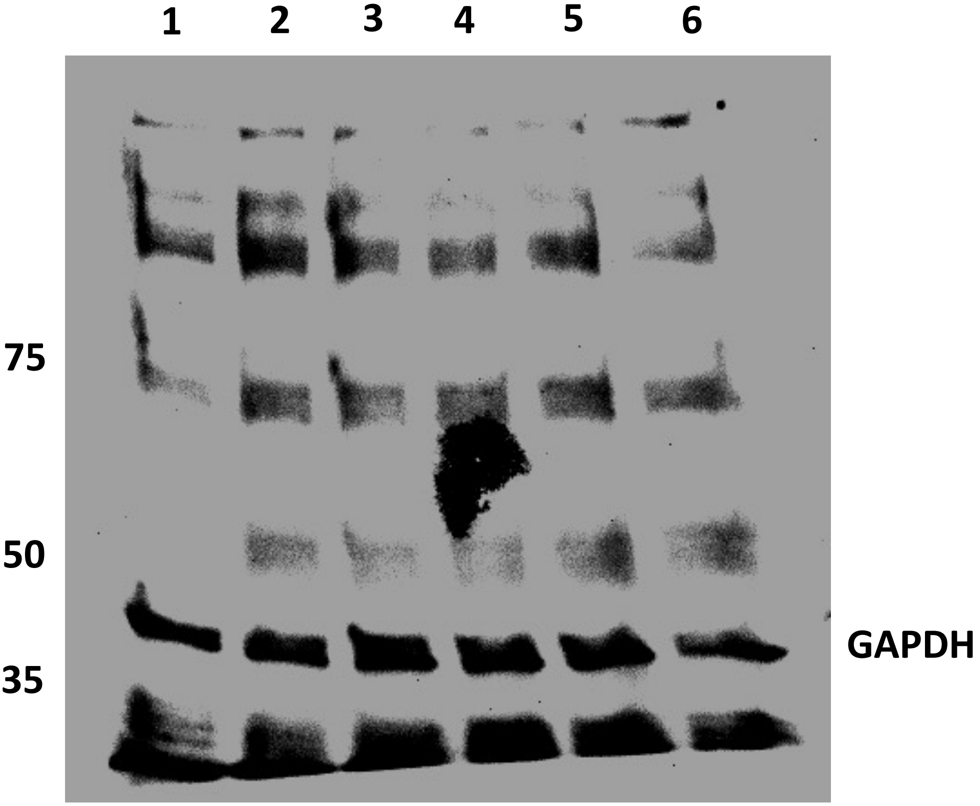

Figure 5C

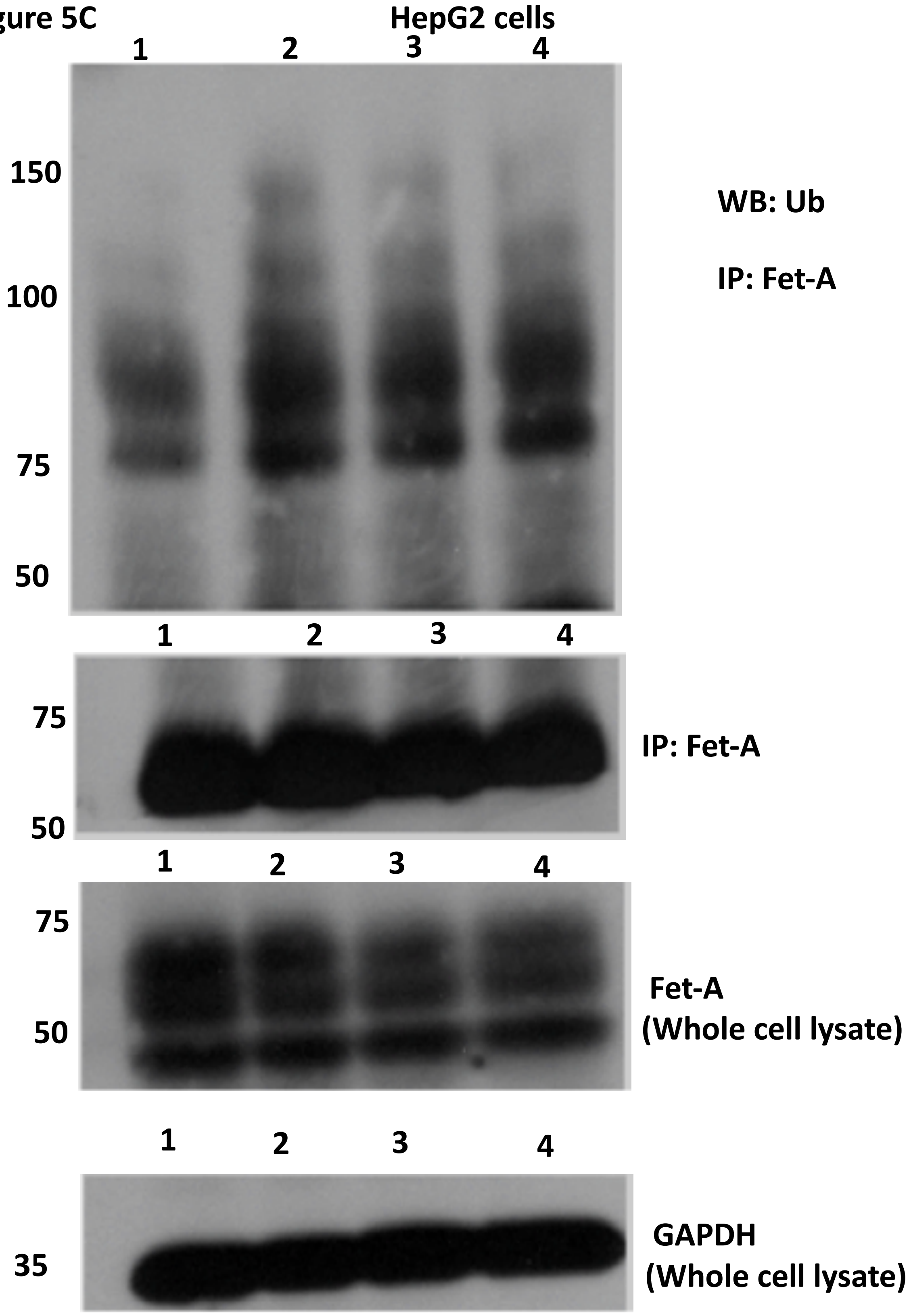
HepG2 cells
1
2
3
4
150
100
75
50
WB: Ub
IP: Fet-A
1
2
3
4
75
50
IP: Fet-A
1
2
3
4
75
50
Fet-A
(Whole cell lysate)
1
2
3
4
35
GAPDH
(Whole cell lysate)

Figure 5D

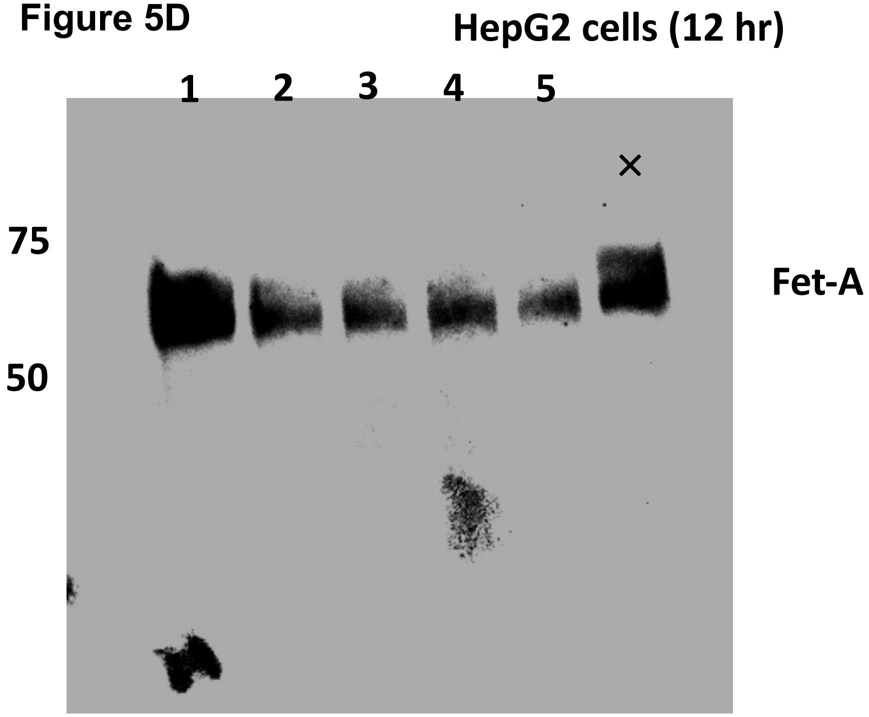

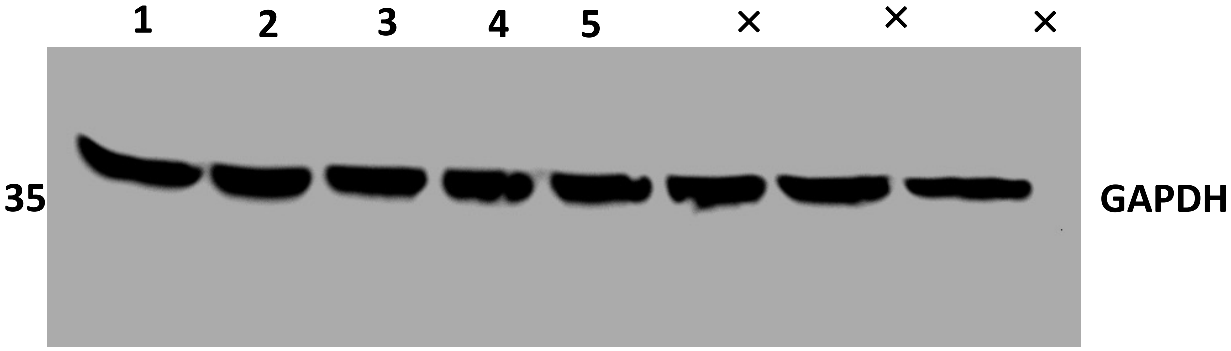

Figure 5F

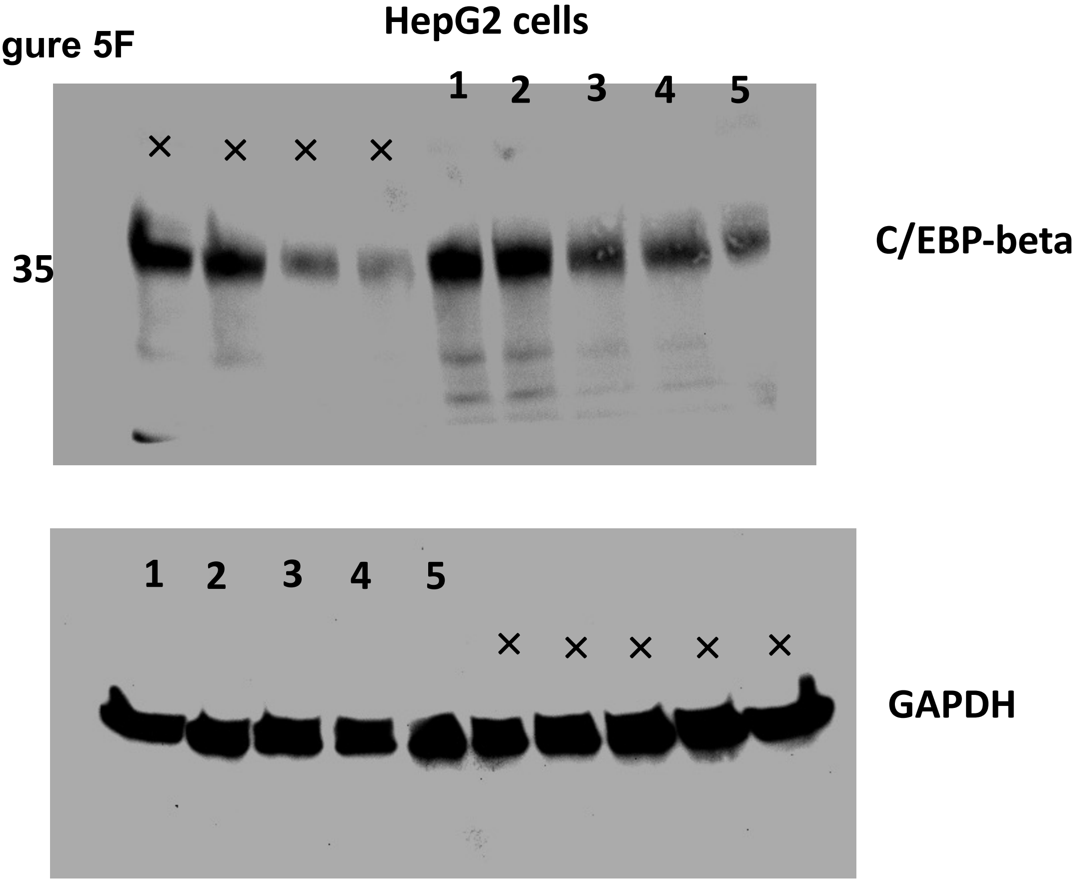

Figure 5H

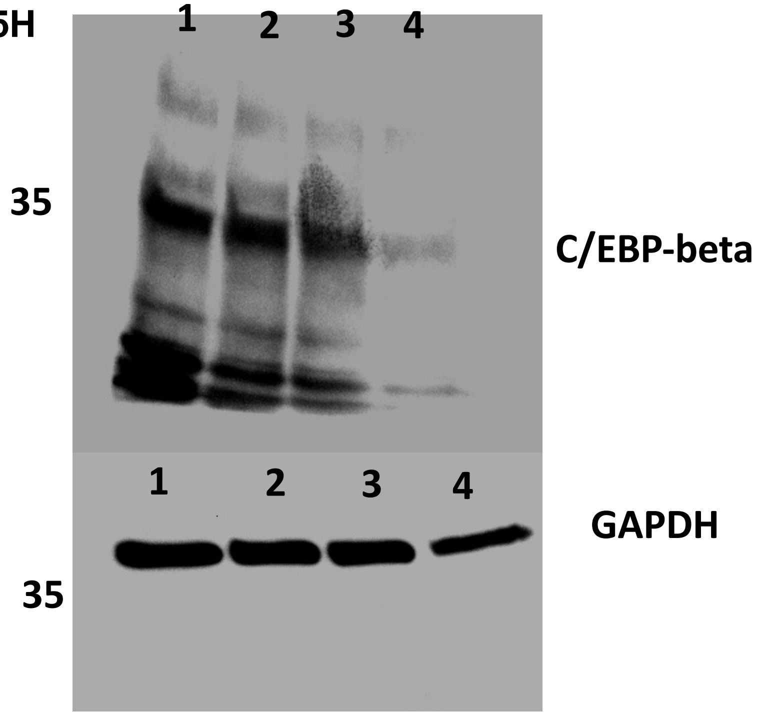

Suppl Figure 1A

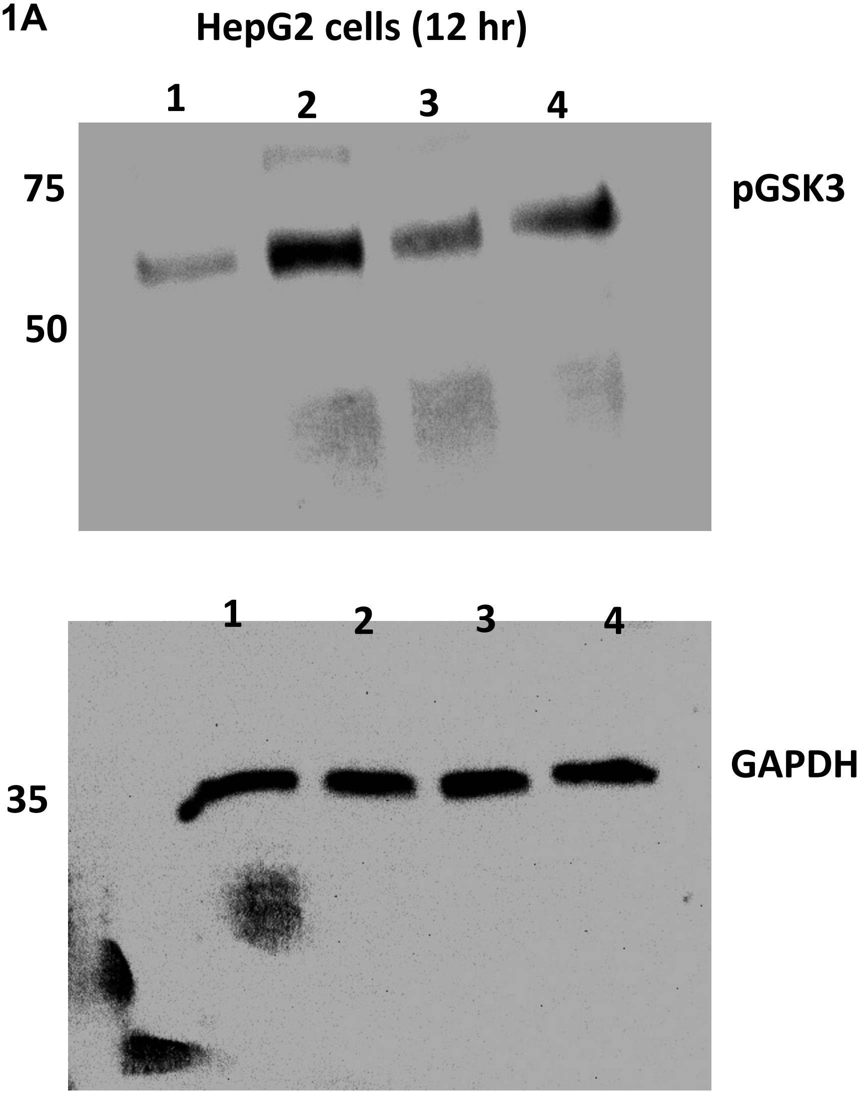

Suppl Figure 1B

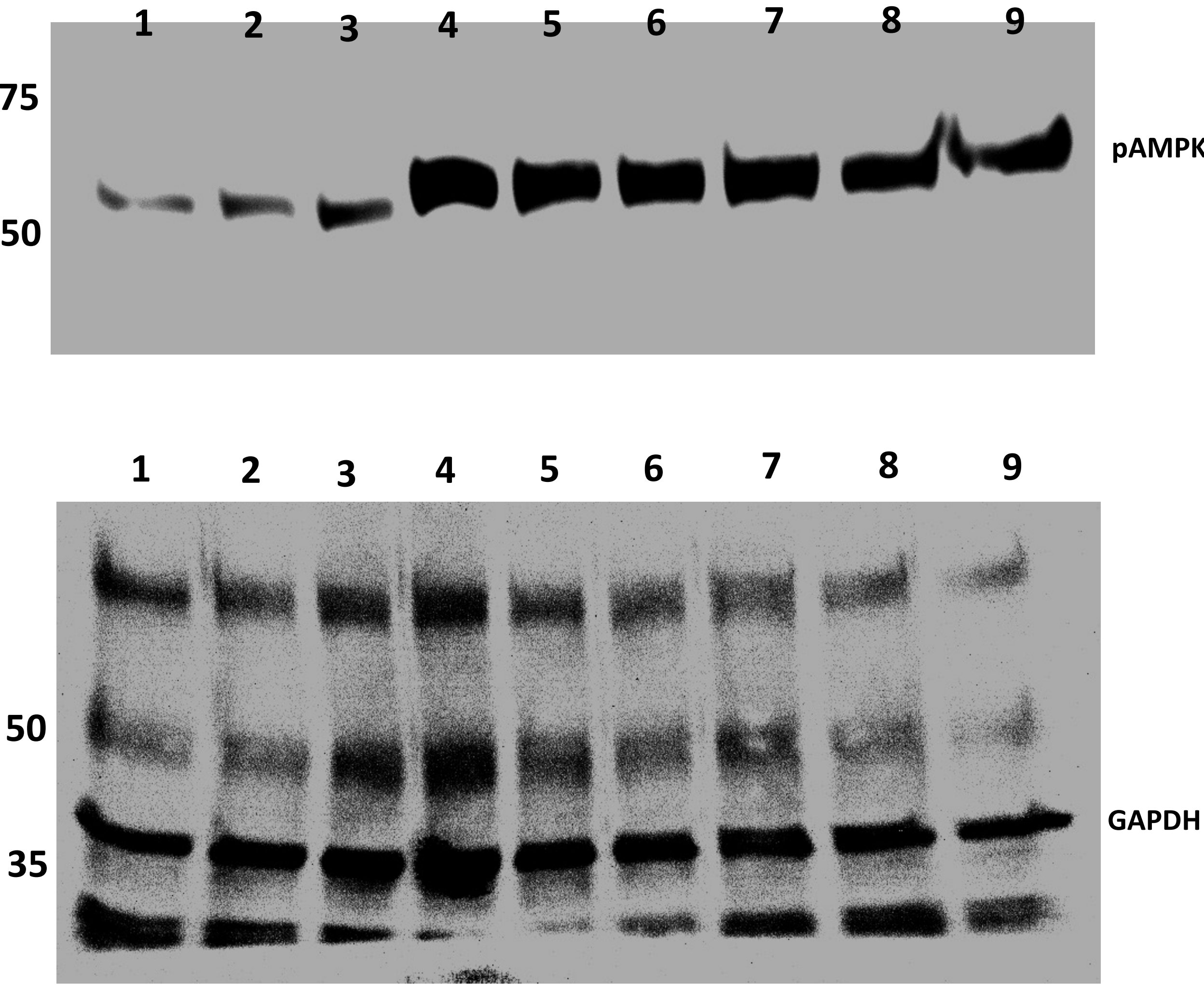

Suppl Figure 1B

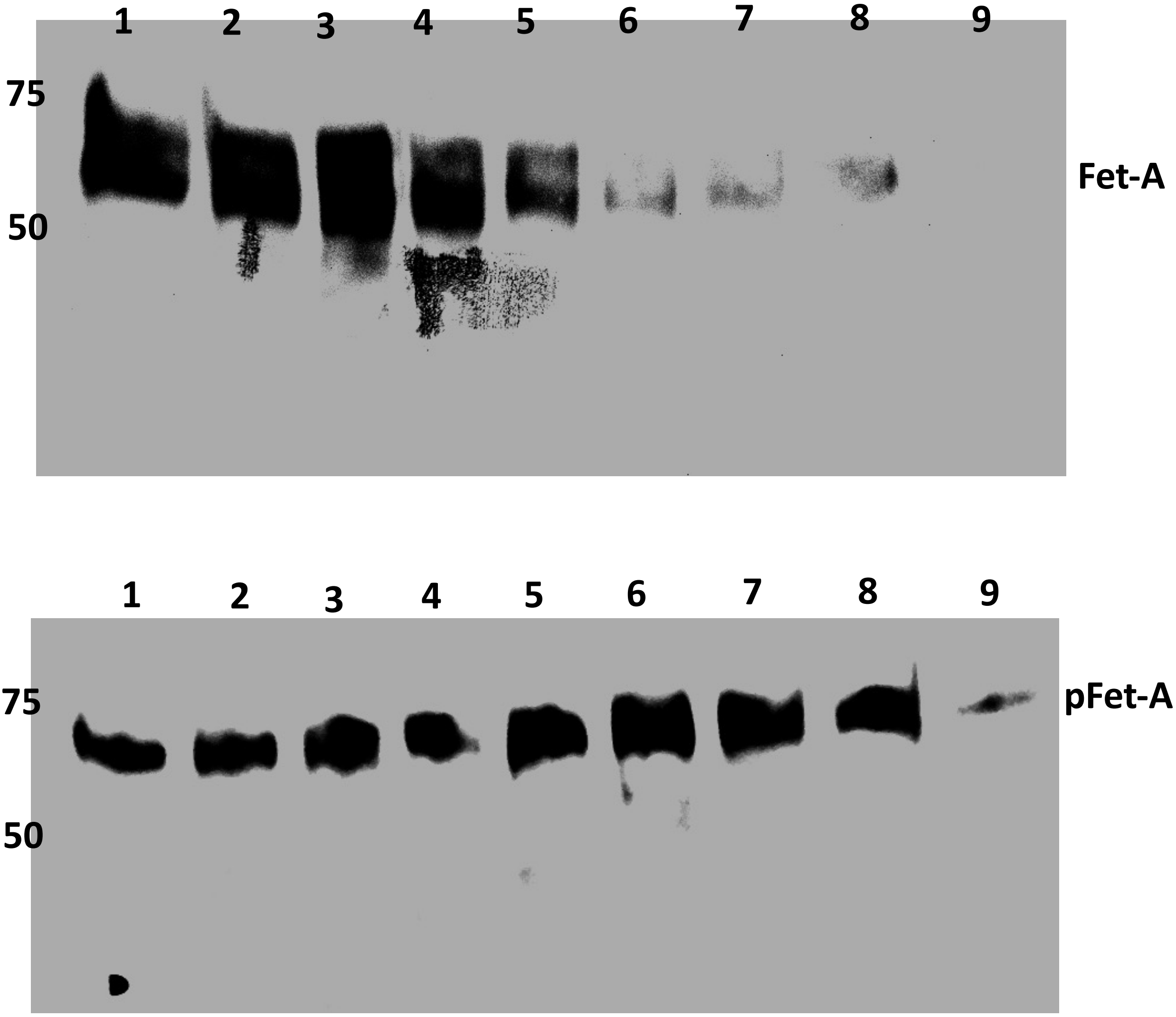

Supplement: S1 Raw images — (PDF) [file pone.0266472.s002.pdf]
